# Effect of the full coverage policy of essential medicines on medication adherence: A quasi-experimental study in Taizhou, China

**DOI:** 10.3389/fpubh.2022.981262

**Published:** 2022-10-12

**Authors:** Zhigang Guo, Zixuan He, Huangqianyu Li, Liguang Zheng, Luwen Shi, Xiaodong Guan

**Affiliations:** ^1^Department of Pharmacy, Peking University School and Hospital of Stomatology, Beijing, China; ^2^International Research Center for Medicinal Administration, Peking University, Beijing, China; ^3^School of Pharmaceutical Sciences, Peking University, Beijing, China

**Keywords:** full coverage policy, essential medicines, medication adherence, free medicines, hypertension and diabetes, chronic diseases, China

## Abstract

**Objective:**

Different forms of full coverage policy of essential medicines (FCPEMs) have been adopted worldwide to lower medication expenditure and improve adherence. This study aims to analyse the effect of FCPEMs on patients' medication adherence in Taizhou city, China.

**Methods:**

This study was a quasi-experimental study and set treatment and control groups. We extracted Electronic Health Records (EHRs) for hypertension and diabetes 1 year before and after FCPEMs implementation and their medication adherence level assessed by physicians. We applied the propensity score matching (PSM) method to balance the bias between the two groups. Then, the descriptive analysis was used to compare the differences in the reported medication adherence. Using the Difference-In-Differences (DIDs) method, the fixed-effect model with the logistic regression was built to analyse the effects of FCPEMs.

**Results:**

225,081 eligible patients were identified from the original database. In the baseline year, FCPEM covered 39,251 patients. After PSM, 6,587 patients in the treatment group and 10,672 patients in the control group remained. We found that the proportion of patients with high adherence in the treatment group increased by 9.1% (60.8 to 69.9%, *P* < 0.001) and that in the control group increased by 2.6% (62.5 to 65.2%, *P* < 0.001). The regression results showed that FCPEMs significantly increased patients' medication adherence (OR = 2.546, *P* < 0.001).

**Conclusion:**

FCPEMs significantly improved medication adherence. Socially disadvantaged individuals might benefit more from continuing FCPEM efforts. Expanding the coverage of FCPEMs to other medicines commonly used in patients with chronic diseases may be a promising strategy to manage chronic diseases and promote patient outcomes.

## Introduction

Medicine cost is one of the leading causes of suboptimal medication adherence and underutilization, aggravating disease burden, especially for chronic diseases. Reducing out-of-pocket expenses for evidence-based therapies proved an effective strategy for promoting rational use of medicines and lower rates of preventable complications (1–3). The full coverage policy was also known as “free,” “full reimbursement,” or “fee exemption” medicines policy. Full coverage policies of essential medicines (FCPEMs) were also put forward to improve the availability and affordability of essential medicines worldwide (4). According to the Pharmaceutical Country Profiles by the World Health Organization, all the 105 listed countries have made free medicines policies, and 54 provided full coverage for various medicines for essential medicine and 56 for chronic diseases (5).

Though FCPEMs implementation varied across countries and regions, many FCPEM programs prioritized vulnerable populations such as children and/or the elderly, patients from lower-income groups, and those with chronic diseases. For instance, Burkina Faso eliminates fees for healthcare utilization for children under five (6). Similar fee-exemption policies for children in primary care settings can be found in the US and Japan (7, 8). Brazilian National Health System provides free access to medicines for older adults in primary care (9), while Spain made prescription medicines free to elderly individuals (10). Other full coverage policies prioritized rural and low-income groups, as seen in France, Canada, and China (11–13). Many such programs also prioritized treatments for hypertension and diabetes due to their high incidence rates and serious consequences related to poor disease control (14–18). For example, the “Farmácia Popular” programme in Brazil made essential oral hypoglycaemic and antihypertensive medicines free to patients in 2011 (19). In the US, the Diabetes Health Plan reduced cost-sharing for metformin, statins, and ACE/ARBs (20). Research on medicine utilization showed that FCPEMs could increase the use of covered medicines and improve patients' overall adherence, though the extent of policy effect varied across studies (16, 19–21).

China bears a heavy burden from cardiovascular and kidney diseases due to complications of hypertension and diabetes (22, 23). Thus, prioritizing chronic disease control can have implications for the control of other diseases. To explore various strategies in chronic disease management, pilot FCPEMs have been launched in selected areas of 16 provinces in China by the end of 2020 (5, 12, 24, 25). However, studies examining the effect of policies on patients' medication adherence are limited. This study aims to analyse the effect of FCPEMs on patients' level of medication adherence with a longitudinal dataset in Taizhou, China, one of the first pilot areas, and to identify strategies to enhance adherence of patients with chronic diseases.

## Methods

### Study setting and policy introduction

Taizhou is a prefecture-level city with a total area of 10,050 km^2^ in Zhejiang Province, located in the central area of the Yangtze River Delta in China. The city administers three urban districts (Jiaojiang, Huangyan, and Luqiao), three county-level cities (Linhai, Wenling, and Yuhuan) and three counties (Tianhai, Xianju, and Sanmen). In 2011, Taizhou had a population of 5.8 million, of which 9.1% were aged over 65 years, 19.3% were aged 45–64 years, and the per capita GDP was 7,287.4 dollars. There were 3,061 health institutions, 156 hospitals and health centers, 29,890 health professionals, 12,606 licensed physicians and 17,536 hospital beds (26).

To promote adherence to medicines and control of chronic disease, at the end of 2011, Taizhou city required all the nine districts and counties within its jurisdiction to establish a catalog between 2012 and 2013, specifying which hypertension and diabetes medicines are listed in China's National Essential Medicines List (version 2012) were to reimburse in full (27, 28). In June 2012, Huangyan was the first district in Taizhou to announce its reimbursement list of hypoglycaemic (metformin hydrochloride and glipizide tablets) and antihypertensive medicines (captopril and indapamide tablets), and all districts implemented their respective FCPEM policies in October 2013. All patients living in Taizhou could access to medicines listed in this catalog without any costs, including drug, prescription and related medical costs, at any primary care or designated facilities. As part of the basic public health services, from the new healthcare reform in 2009, China has established health records and provided free chronic disease management services for hypertension and diabetes (29, 30). According to the FCPEMs of Taizhou, physicians at primary care and designated facilities were responsible for maintaining health records of patients, providing regular follow-ups, recording and reporting the medicines' clinical benefits, evaluating appropriateness for the patient and adjusting the medicine plan, such as quitting free medicines if clinical outcomes were poor. All patients' prescriptions benefiting from the FCPEMs were integrated into their Electronic Health Records (EHRs), which gather local patients' health records, including demographics, diagnosis and disease profile, medicine use, and health behaviors.

### Study design

This study was a quasi-experimental study, which set treatment and control groups and used the longitudinal EHRs data to examine the effect of FCPEMs policy on patients' medication adherence. We controlled for confounding factors between two groups using the difference-in-differences (DIDs) method (31) and analyzed the difference in changes in medication adherence between the two groups 1 year before and after the policy implementation. Huangyan district was identified as the treatment group as it was the first to implement the FCPEMs in Taizhou. Linhai and Wenling were regarded as the control group. We applied the propensity score matching (PSM) using nearest neighborhood matching to eliminate possible influences of substantial baseline differences between groups, ensuring a more rational interpretation of the causal effect. We applied a caliper of 10^−6^ to reduce the matching tolerance and allowed equally qualified objects to retain in this step.

We conducted analysis on both the whole and the matched samples to ensure the stability of the results.

### Data source and study population

**Figure 1:**
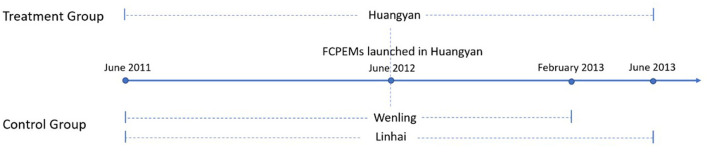
Study timeline. Wenling implemented the FCPEMs in February 2013. Linhai launched its respective policy in October 2013, which is beyond the study period.

We extracted EHR data from Taizhou's database for chronic disease management. Due to a system upgrade, data from six districts were inaccessible and thus excluded from our study. Therefore, only Linhai and Wenling were altogether taken as the control group. We defined records collected from Huangyan, Wenling and Linhai between June 2011 and June 2012 as the baseline data. As Wenling and Linhai announced the FCPEMs in February and October 2013, we defined follow-up data as records from June 2012 to June 2013 in Huangyan and Linhai, and from June 2012 to February 2013 in Wenling. We subsequently established a 2-year cohort dataset. Figure 1 illustrates details of the study timeline.

### Outcome measure

According to the requirements and regulations of the Chronic Disease Management Services of China, family physicians should administer follow-up surveys regularly and file follow-up records into EHRs. The EHRs system categorizes adherence indicators into “regular medicine use,” “interrupted medicine use,” or “taking no medicine” at each follow-up. In this analysis, “regular medicine use” was identified as high adherence (=1) while “irregular medicine use” and “taking no medicine” were regarded as poor adherence (=0).

Besides the primary variable of concern, categorical variables, including the patient's characteristics, socioeconomic status, and health behaviors, were identified as controls and used for PSM (Table 1). These included the patient's age, gender, insurance scheme, annual average BMI index, marital status, monthly household income per person, employment status, educational attainment, smoking and drinking habit, and hypertension and diabetes history.

**Table 1:** Characteristics of study population before and after PSM.

**Variables**	**Description**	**Before PSM**			**After PSM**		
		**Treatment** **(*n* = 39,251)**	**Control** **(*n* = 185,830)**	***P***	**Treatment** **(*n* = 6,587)**	**Control** **(*n* = 10,672)**	***P***
Medication adherence	High adherence	48.5%	51.1%	0.000	62.5%	60.8%	0.043
	Poor adherence	51.5%	48.9%		37.5%	39.2%	
Gender	Female	62.7%	59.6%	0.000	70.4%	70.1%	0.703
	Male	37.3%	40.4%		29.6%	29.9%	
Age, years	0–64	51.0%	49.1%	0.000	54.3%	55.0%	0.473
	≥65	49.0%	50.9%		45.7%	45.0%	
Household monthly income per person, CNY^a^	≤ 500	11.5%	8.9%	0.000	8.7%	7.8%	0.586
	500–3,000	51.0%	38.9%		47.5%	47.9%	
	≥3,000	37.5%	52.2%		43.8%	44.3%	
Residential terrain	Plain area	80.4%	73.2%	0.000	90.1%	88.6%	0.005
	Mountainous area	19.6%	26.8%		9.9%	11.4%	
Marital status	Married^b^	83.1%	80.8%	0.000	85.2%	85.9%	0.265
	Single^c^	16.9%	19.2%		14.8%	14.1%	
Employment status	Employed	5.9%	17.0%	0.000	3.5%	2.6%	0.006
	Unemployed^d^	94.1%	83.0%		96.5%	97.4%	
Education attainment	Illiterate	34.5%	45.8%	0.000	40.1%	39.8%	0.530
	Primary school	45.2%	39.9%		47.0%	47.1%	
	Secondary school	17.0%	11.9%		11.8%	11.8%	
	High school and above	3.3%	2.4%		1.2%	1.3%	
Insurance	None	2.5%	14.2%	0.000	2.0%	2.0%	0.203
	URRBMI^e^	93.1%	84.3%		97.5%	97.6%	
	UEBMI/CMI^f^	4.4%	1.5%		0.5%	0.4%	
BMI, kg/m^2^	Average BMI	23.1	23.4	0.000	23.1	23.0	0.732
Smoking	Yes	18.5%	2.6%	0.000	3.2%	2.7%	0.078
	No	81.5%	97.4%		96.8%	97.3%	
Drinking	Yes	12.3%	1.6%	0.000	1.9%	1.9%	0.898
	No	87.7%	98.4%		98.1%	98.1%	
Disease	Hypertension	81.6%	83.9%	0.000	74.4%	74.4%	1.000
	Diabetes	18.4%	16.1%	0.000	25.6%	25.6%	

a CNY, Chinese yuan.

b Married including married and remarried.

c Single including unmarried, divorced, and widowed.

d Unemployed including unemployed and rural residents.

e URRBMI, Urban-rural resident basic medical insurance.

f UEBMI/CMI, Urban employee basic medical insurance or/and commercial medical insurance.

### Statistical approaches

The study conducted a descriptive analysis to indicate the impacts of the FCPEMs on medication adherence, with Student's *T* Test for continuous variables and Chi-square Test for categorical variables. We constructed a fixed-effect model with logistic regression would be constructed to ascertain the statistical significance of the findings, ensuring the scientific and rigorous interpretation of our study results. The DIDs method is incorporated into the fixed-effect model to control for heterogeneity. The analytic model was constructed as:


(1) $$\begin{array}{l} \begin{array}{lll} logit\;Y_{it} & = & \beta_{0}+\beta_{1}\;Group_{i}\times Time_{t}+\beta_{2}\;Group_{i}+\beta_{3}\;Time_{t} \end{array}\\ \;+\beta \;X_{it}+\varepsilon_{it} \end{array}$$

Where *Y*_*it*_ denoted medicine adherence of every individual at different times, *Group* represented the individual's participation category (=1 if in the treatment group, = 0 if in the control group), *Time* indicated the stage of policy implementation (=1 if after policy implementation, =0 if prior to implementation), β_0_ was the regression intercept, and ε_*it*_ denoted an idiosyncratic error that changed across individuals and time. *X*_*it*_ captured other individual and household characteristics for control. The coefficient of interest β_1_ gave the estimate of the average treatment effect of FCPEMs on medication adherence. The coefficients were interpreted in terms of odds ratio (OR).

All analyses were programmed in STATA 14.0.

## Results

### Characteristics of study population

225,081 patients and their respective records were included in the baseline year. Among them, 39,251 patients were covered by FCPEMs. Table 1 showed that all variables of concern differed significantly at the 0.001 significance level between the treatment and control groups of the original cohort. After PSM, 6,587 and 10,672 patients remained in the treatment and control groups, respectively. Baseline differences in most variables were eliminated at the 0.05 level, except for medication adherence (*P* = 0.043), residence (*P* = 0.005), and employment status (*P* = 0.006).

### Description of changes in medication adherence

As indicated in Table 2, the sample population increased from 225,081 to 267,854 patients during the study period, while the cohort size after PSM slightly decreased from 17,259 to 16,842 patients. The proportion of patients with high adherence without matching increased by 5.3% (51.1–56.4%, *P* < 0.001) among the control group and 18.5% (48.5–67.0%, *P* < 0.001) among the treatment group. After matching, the proportion of patients with high adherence in the control group increased by 2.6% (62.5–65.2%, *P* < 0.001) and that in the treatment group increased by 9.1% (60.8–69.9%, *P* = *P* < 0.001). The results showed that FCPEMs could promote medication adherence.

**Table 2:** The proportion of patients with high adherence before and after FCPEMs.

**Group**	**Before PSM**			**After PSM**		
	**Before FCPEMs**	**After FCPEMs**	**Diff**	**Before FCPEMs**	**After FCPEMs**	**Diff**
Control group	51.1%	56.4%	5.3%^***^	62.5%	65.2%	2.6%^***^
	(*n* = 185,830)	(*n* = 216,735)		(*n* = 10,672)	(*n* = 10,412)	
Treatment group	48.5%	67.0%	18.5%^***^	60.8%	69.9%	9.1%^***^
	(*n* = 39,251)	(*n* = 51,119)		(*n* = 6,587)	(*n* = 6,430)	
ΔDiff			13.2%			6.5%

*** *P* < 0.001.

### Regression analysis on the effects of FCPEMs

As the fixed-effect model omitted all time-invariant covariates, only Group × Time (policy indicator), Time, Age, BMI, Smoking and Drinking were retained in the results. Table 3 shows the results of the fixed-effect regression analysis before and after PSM. FCPEMs had a significant positive impact on patients' medication adherence in both the original sample (OR = 2.825, *P* < 0.001) and the sample after PSM (OR = 2.546, *P* < 0.001). That means the treatment group had 2.825 times more likely to be high adherence than the control group in the original sample and 2.546 times in the sample after PSM. Patients' medication adherence experienced a natural increase with the progression of time in samples without PSM (OR = 2.285, *P* < 0.001) and with PSM (OR = 1.647, *P* < 0.001). Moreover, results from the unmatched sample indicated that the patient's BMI level had a significant positive association with adherence (OR = 1.033, *P* = 0.009). Other factors present no statistically significant effects on the outcome.

**Table 3:** Fixed-effect regression analysis of the effect of FCPEMs on medication adherence.

**Variables**	**Before PSM**		**After PSM**	
	**OR value (95% CI)^a^**	***P***	**OR value (95% CI)**	***P***
Group × Time-FCPEMs	2.825 (2.567–3.099)	0.000	2.546 (2.028–3.197)	0.000
Time, year	2.285 (2.186–2.388)	0.000	1.647 (1.404–1.933)	0.000
Age ≥ 65	1.061 (0.810–1.389)	0.667	1.900 (0.601–6.006)	0.274
BMI, kg/m^2^	1.033 (1.008–1.059)	0.009	0.998 (0.920–1.083)	0.962
Smoking-Yes	0.864 (0.739–1.010)	0.066	1.434 (0.682–3.014)	0.342
Drinking-Yes	0.869 (0.712–1.060)	0.166	0.673 (0.301–1.505)	0.335

a OR, odds ratio; CI, confidence interval.

## Discussion

This study found that FCPEMs could significantly promote medication adherence based on patient-level data. The proportion of patients with high level of medication adherence increased by 13.2% (18.5–5.3%) in the unmatched population and by 6.5% (9.1–2.6%) after matching. The fixed-effect model further suggested that the policy effect was statistically significant, which aligns with results from previous literature (16–21, 32). Yet, FCPEMs in Taizhou covered only four hypertensive and diabetic medicines, which might not meet the complex and diverse needs of patients (2). Future policy design should target medication adherence and consider expanding the success of pilot FCPEMs interventions across the system with considerations of treatment algorithms. For example, Brazilian ‘Farmácia Popular’ offered 17 kinds of hypertensive and diabetic medicines for free and achieved remarkable adherence improvement (19) while the US Diabetes Health Plan covering only three medicines only showed a modest adherence rise (20).

Furthermore, many policy beneficiaries in our cohort sample were vulnerable populations, which have implications for studies in disadvantaged settings. Studies have revealed that socially disadvantaged groups demonstrated a lower adherence to medicine due to costs (5, 12, 25). Suboptimal medication adherence further compromises patient health due to increased risk of disease complications, aggravates the burden of disease control, and increases overall healthcare costs. In our study, 19.6% of the treatment group and 26.8% of the control group were residents in remote mountainous areas and unemployment and illiteracy rates were high, with 94% of the treatment group unemployed. 62.4% of the entire sample cohort had a households' monthly income per diem of <3,000 yuan (475.25 USD) and 11.5% had that of even <500 yuan (79.21 USD), which was in stark contrast to Taizhou's average monthly per capita income of 3917.25 yuan (620.55 USD) in 2012. 93.1% of the patients benefiting from FCPEM were enrolled in the URRBMI scheme, which had poor coverage for treatments of chronic diseases. URRBMI beneficiaries with chronic diseases thus might experience lower access to medicines needed and compromised health (33, 34). FCPEMs can be an effective strategy to promote equitable access to medicines, promote patient health, and safeguard vulnerable populations from financial burdens due to medical costs. The Chinese Bureau of Statistics stated that by 2020, 551.62 million population would reside in rural areas of China, and more than 190.45 million people would be employed in the primary industry with an average monthly income of <3,000 CNY (434.78 USD). The World Bank stated that 8.6% of the world population lived under extreme poverty (<59 USD per month), and 1.3 billion people lived in households with multiple layers of deprivations (35). Our findings add to the case for prioritizing the implementation of FCPEMs to improve adherence and alleviate economic and disease burdens.

Our study, however, is subject to several limitations. First, though randomized controlled study design remains the golden standard in examining the effect of the interventions, the assignment to FCPEM in this study was not rigorously randomized. We controlled for the bias with PSM and the fixed-effect model constructed with the DIDs method and subsequently formed a quasi-randomized controlled study design. Still, there were likely other influential factors of medication beyond our scope (36, 37). Second, medication adherence in filed records was assessed by the patient's self-reported adherence. This might have introduced biases. Third, though we aimed to minimize the impact of sample selection on the study outcome by improving the sample representativeness with more generalized policy beneficiaries in this study, whether our study findings are generalisable to other diseases awaits further justifications.

## Conclusion

This study found that FCPEMs is an effective strategy to improve adherence to medicines for chronic diseases, with PSM controlling for baseline biases and the fixed-effect model eliminating time-invariant unobservable factors. For patients with hypertension and diabetes, FCPEMs in Taizhou resulted in a substantial increase in the level of adherence to antidiabetic and antihypertensive medicines. Meanwhile, as our treatment group was mostly vulnerable populations, FCPEMs could be a promising strategy to protect socially disadvantaged groups. Policymakers should consider reducing or removing cost-sharing for essential medicines for chronic diseases.

## Data availability statement

The data analyzed in this study is subject to the following licenses/restrictions: The data used in the study are non-public electronic health records belonging to the health department of Taizhou city, Zhejiang. The data contains patients' personal information, so other researchers need permission from Taizhou city's health department to access the data. Some detailed statistical data underlying this article will be shared on reasonable request to the corresponding author. Requests to access these datasets should be directed to guanxiaodong@pku.edu.cn.

## Ethics statement

The studies involving human participants were reviewed and approved by Peking University Institutional Review Board. Written informed consent for participation was not required for this study in accordance with the national legislation and the institutional requirements.

## Author contributions

ZG, XG, and LS conceptualized and designed the study and contributed to data collection. ZG and LZ participated in data analysis. ZG, ZH, and XG conducted the final analyses and contributed to the interpretation of the results. ZG, ZH, XG, and LS drafted the initial manuscript. All authors have read and agreed to the published version of the manuscript.

## Funding

This study was supported by the National Natural Science Foundation of China (72104011 and 71774005). The Foundations had no role in the study design, data collection, data analysis and interpretation, writing of the manuscript, and the decision to publish.

## Conflict of interest

The authors declare that the research was conducted in the absence of any commercial or financial relationships that could be construed as a potential conflict of interest. The reviewer DZ declared a shared affiliation with the authors to the handling editor at the time of the review.

## Publisher's note

All claims expressed in this article are solely those of the authors and do not necessarily represent those of their affiliated organizations, or those of the publisher, the editors and the reviewers. Any product that may be evaluated in this article, or claim that may be made by its manufacturer, is not guaranteed or endorsed by the publisher.
